# Numerous onchocercomas in an elderly Ugandan woman: A rare presentation of onchocerciasis

**DOI:** 10.1016/j.jdcr.2025.09.001

**Published:** 2025-09-18

**Authors:** Mohamed Jayte, Stephen Mirembe, David Saria Elia, Lubega Athanus, Farah Dubad Abdi, Abukar Ali Ahmed, Adan Abdi Hassan

**Affiliations:** a Internal Medicine Department at Kampala International University, Kampala, Uganda; b Dermatology Department at Kampala International University, Kampala, Uganda

**Keywords:** doxycycline, elderly patient, ivermectin, onchocerciasis, onchocercomas, river blindness, Uganda

## Introduction

Onchocerciasis, or “river blindness,” is a neglected tropical disease caused by *Onchocerca volvulus*, transmitted through blackfly bites. The disease is transmitted by the blackfly (Simulium damnosum), which breeds near fast-flowing rivers.^1^ It causes chronic skin lesions, severe itching, and blindness, significantly impacting morbidity in endemic regions. Globally, 20.9 million people are infected, with 99% of cases in sub-Saharan Africa.^2^ Uganda, particularly its northern and western regions near the Nile, the disease is highly endemic. Despite mass drug administration (MDA) programs using ivermectin, challenges like limited health care access and population displacement sustain.^3^

Onchocercomas, subcutaneous nodules containing adult worms, are a hallmark of chronic infection. While typically found on bony prominences, multiple widely distributed nodules are rare, especially in elderly patients with long-term exposure. Uganda predominantly reports the nonblinding form of onchocerciasis, in contrast to West Africa, where the blinding variant is more prevalent. Such cases are often misdiagnosed, delaying treatment and increasing complications.^4^ In addition to physical symptoms, patients may experience significant psychosocial morbidity due to visible skin lesions and stigmatization. Nodding syndrome, a seizure disorder linked to Onchocerca volvulus, has also been reported in parts of Uganda.

This case report describes an elderly patient with widespread onchocercomas, a rare manifestation of onchocerciasis.

## Case presentation

**Fig 1:**
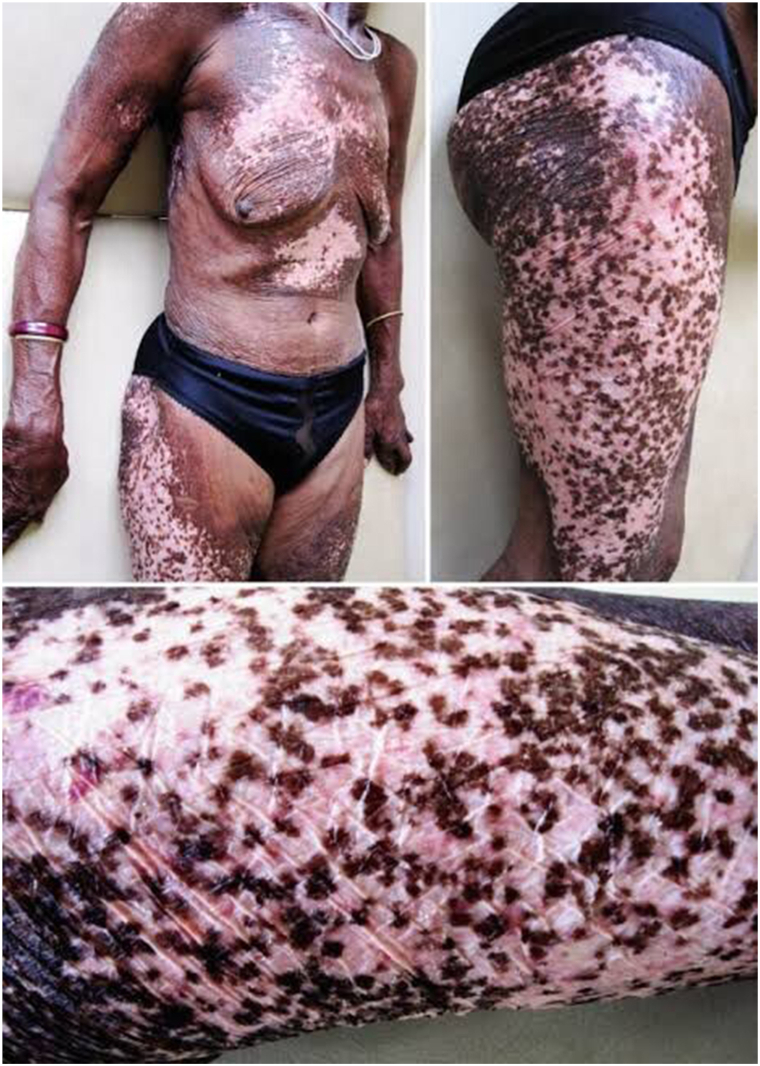
Generalized onchodermatitis with hyperpigmented papules and plaques.

A 62-year-old female farmer from western Uganda presented with multiple, painless, subcutaneous nodules that had grown over 15 years, increasing in size and number in the past 5 years. She reported occasional pruritus but no systemic symptoms. She first noticed pigmentary skin changes approximately 5 years ago, which coincided with a noticeable increase in the number and size of nodules. Living near the Mpanga River, she had long-term exposure to blackfly bites but had never received ivermectin due to limited health care access. Physical examination revealed firm, nontender nodules (1-5 cm) on the iliac crests, ribs, knees, and scapulae, with hyperpigmentation, patchy hypopigmentation, and lichenification consistent with chronic onchocercal dermatitis (Fig 1). On examination, over 15 nodules were palpated, ranging from 1.2 to 5.0 cm. Typically, patients in endemic areas develop 3-6 nodules, making this case unusually severe. No ocular involvement or systemic abnormalities were noted. The patient and her community were unaware of onchocerciasis and its manifestations. The village had not been included in previous ivermectin MDA campaigns due to poor health care access and logistical challenges.

**Fig 2:**
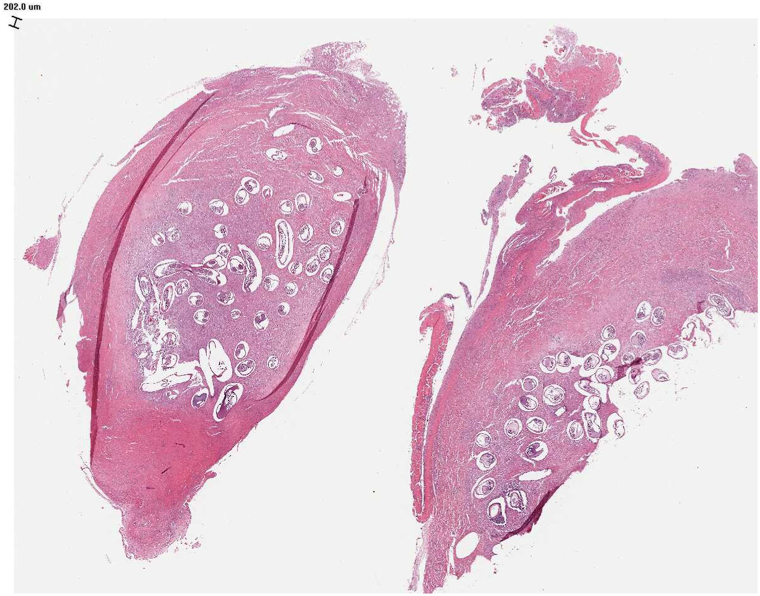
Histopathology of an onchocercoma (hematoxylin and eosin stain).

Laboratory tests showed mild eosinophilia (12%, 1200 cells/μL), and enzyme-linked immunosorbent assay confirmed *Onchocerca volvulus* antibodies. Ultrasound revealed hypoechoic masses; fine needle aspiration was initially performed but definitive diagnosis was established via histopathology of excised nodules. Treatment included ivermectin (150 mcg/kg) every 6 months, a 6-week course of doxycycline (100 mg daily), antihistamines for pruritus, and surgical excision of 2 large nodules. Doxycycline was used to target Wolbachia, an endosymbiotic bacterium critical for the survival and fertility of adult O. volvulus. Elimination of Wolbachia results in worm sterility and gradual death, enhancing long-term treatment outcomes. Histopathology confirmed the presence of adult worms (Fig 2). The excised nodules measured up to 4.8 cm. Histological cross-sections revealed multiple adult worms, including females with gravid uteri and embedded microfilariae, surrounded by fibrous tissue.

At follow-up, the patient reported reduced pruritus and no new nodules. Serological titers declined, indicating reduced parasite burden, and ophthalmologic evaluation remained normal. She continued annual ivermectin treatment and community-based MDA programs to prevent reinfection.

## Discussion

This case report describes a rare presentation of multiple, widely distributed onchocercomas in an elderly patient from western Uganda, an area endemic for onchocerciasis. The findings highlight the chronic nature of *Onchocerca volvulus* infection and the challenges of diagnosing and managing advanced disease in resource-limited settings. The patient presented with multiple subcutaneous nodules, chronic skin changes, and mild eosinophilia, consistent with long-standing onchocerciasis, a major public health issue in sub-Saharan Africa.^1^

Onchocerciasis, transmitted by blackflies, causes skin disease and blindness. Microfilariae migrate to the skin and eyes, causing inflammation, while adult worms in nodules can survive for years, leading to complications like widespread nodule formation.^2^ Although repeated exposure to blackfly bites is common in endemic regions, most individuals develop fewer than 5-6 nodules. The presence of over 15 onchocercomas in this patient indicates a long-standing, untreated infection, which is uncommon, especially among elderly patients. Onchocercomas are typically subcutaneous and found over bony prominences, but studies show that deeper nodules may be present and not clinically palpable. The patient’s long-term exposure to blackfly-infested areas and lack of ivermectin treatment likely contributed to disease progression. Although repeated exposure to blackflies can lead to infection, most individuals develop only a few onchocercomas. Multiple large nodules in a single patient, as observed here, are rare and indicate chronic, untreated infection.

Diagnosis was confirmed through clinical findings, serological tests, and histopathology. However, limited access to advanced diagnostics in Uganda often leads to delayed or misdiagnosis, especially in atypical cases. Differential diagnoses, including lipomas and tuberculosis, were ruled out through laboratory and imaging studies.^3^

Treatment included ivermectin to kill microfilariae, doxycycline to target *Wolbachia* bacteria in adult worms, and surgical excision of large nodules. The patient showed significant improvement during follow-up, with reduced pruritus and no new nodules.^4^

Although onchocerciasis is known to cause blindness, this patient had no ocular involvement. Uganda predominantly reports the nonblinding form of Onchocerca volvulus, in contrast to West Africa, where the blinding variant is more prevalent. However, the exact reasons behind these regional differences in clinical manifestation remain unclear and require further research.^4^

This case aligns with studies emphasizing the challenges of managing onchocerciasis in endemic regions. Murdoch et al reported similar cases in Nigeria, while Basáñez et al highlighted the benefits of combining ivermectin and doxycycline.^2^^,^^5^ Sustained efforts, including MDA and community education, are crucial to reducing the disease burden in Uganda.^6^ Despite the subtlety of clinical findings in the photograph, the visual documentation adds value by demonstrating the dermatological manifestations of chronic onchocerciasis.

## Conclusion

This case demonstrates a rare presentation of numerous onchocercomas in an elderly woman from an endemic region in Uganda, highlighting the potential for long-standing, untreated onchocerciasis in underserved communities. Despite national MDA efforts, gaps in health care access can result in significant disease progression. The presence of over 15 nodules, confirmed by clinical examination, ultrasound, and histopathology, underlines the importance of maintaining vigilance for atypical presentations, even in well-known endemic zones. Comprehensive approaches that combine early diagnosis, ivermectin and doxycycline therapy, surgical management, and improved community outreach are essential. Future research should also explore the psychosocial impact and hidden disease burden, particularly in remote and neglected populations.

## Conflicts of interest

None disclosed.

## References

[1] Pierre K., Flore N.N., Archile P., Alfons R. (2025). Knowledge and practices of four onchocerciasis-endemic communities in Cameroon. Microorganisms.

[2] Basáñez M.G., Pion S.D.S., Churcher T.S., Breitling L.P., Little M.P., Boussinesq M. (2006). River blindness: a success story under threat?. PLoS Med.

[3] Higazi T.B., Zarroug I.M., Mohamed H.A. (2013). Interruption of Onchocerca volvulus transmission in the Abu Hamed focus, Sudan. Am J Trop Med Hyg.

[4] Katabarwa M.N., Habomugisha P., Agunyo S. (2010). Traditional kinship system enhanced classic community-directed treatment with ivermectin (CDTI) for onchocerciasis control in Uganda. Trans R Soc Trop Med Hyg.

[5] Murdoch M.E., Asuzu M.C., Hagan M. (2002). Onchocerciasis: the clinical and epidemiological burden of skin disease in Africa. Ann Trop Med Parasitol.

[6] Garms R., Lakwo T.L., Ndyomugyenyi R. (2009). The elimination of the vector Simulium neavei from the Itwara onchocerciasis focus in Uganda by ground larviciding. Acta Trop.

